# Circulating Cytokines Mediate the Protective Effect of Physical Activity on Cardiovascular Diseases: A Mendelian Randomization Mediation Analysis

**DOI:** 10.3390/ijms26104615

**Published:** 2025-05-12

**Authors:** Yulin Sun, Yining Liu

**Affiliations:** Department of Sports Science, Hanyang University ERICA, Ansan 15588, Republic of Korea; syl0312@hanyang.ac.kr

**Keywords:** circulating cytokines, physical activity, cardiovascular diseases, Mendelian randomization

## Abstract

Cardiovascular diseases (CVDs) represent a major public health concern globally, being one of the leading causes of illness and death across populations. While physical activity is widely recognized as a protective factor against these diseases, the exact biological mechanisms that explain this relationship are still not fully understood. This study utilized a two-sample Mendelian randomization (MR) method to investigate the potential role of circulating cytokines in mediating the effects of physical activity on major CVD outcomes, including coronary artery disease (CAD), heart failure (HF), and ischemic heart disease (IHD). Our primary MR analysis revealed an inverse association between physical activity and IHD risk. Moreover, a two-step MR mediation approach identified IL10RB (Interleukin-10 receptor subunit beta) as an intermediate mediator, explaining about 6.65% of the observed contribution of physical activity to IHD. These results indicate that physical activity may mitigate CVD risk through modulation of immune pathways, particularly via IL10RB signaling. Our findings underscore the significance of cytokine networks in mediating the cardiovascular benefits of exercise and suggest potential therapeutic strategies for CVD risk reduction through immune system modulation.

## 1. Introduction

Cardiovascular diseases (CVDs) are among the major contributors to global health burdens, accounting for a significant proportion of mortality and disability worldwide [1,2]. Physical activity is widely recognized as a modifiable factor that can reduce the risk of CVD. Numerous epidemiological and clinical studies have consistently reported an inverse relationship between regular physical activity and the occurrence of coronary artery disease (CAD), heart failure (HF), and ischemic heart disease (IHD)—three major forms of CVD [3,4,5]. However, the question of causality remains unresolved. Observational studies can be confounded by lifestyle or socioeconomic factors, and they may be influenced by reverse causation, where individuals with preclinical disease reduce their physical activity levels rather than inactivity leading to disease [6].

Recent developments in immunology and molecular cardiology have proposed that systemic inflammation might mediate the impact of physical activity on CVD risk [7]. Circulating cytokines, including interleukins, tumor necrosis factors, and chemokines, contribute to the inflammatory cascade that promotes atherosclerosis, endothelial dysfunction, and cardiac remodeling [8]. Elevated levels of these inflammatory cytokines exacerbate plaque formation and destabilization, contributing to the worsening of cardiovascular disease [9]. However, emerging evidence indicates that regular physical activity may mitigate these inflammatory pathways by regulating cytokine levels, potentially offering protective effects against disease progression [10]. In addition, research has highlighted the role of cytokines in various chronic diseases, including autoimmune disorders and cancer, through their involvement in immune regulation and inflammation [11,12]. Despite this, the exact mechanisms through which physical activity affects cytokine profiles remain unclear.

Establishing causality between physical activity and cytokine modulation is challenging. Traditional observational designs struggle to determine whether physical activity itself drives changes in cytokine levels or if individuals with healthier immune profiles are simply more likely to engage in physical activity. Additionally, residual confounding factors often limit the clarity of causal interpretations [13]. Mendelian randomization (MR) offers an effective solution by using genetic variants as instrumental variables (IVs), reducing the influence of confounding and reverse causality biases [14,15].

When coupled with mediation analysis, MR offers further insights into the mechanisms underlying these associations. Specifically, MR mediation analysis allows for the estimation of the proportion of an exposure’s effect on a result that is conveyed through an intermediary [16]. This method enables a clearer understanding of how lifestyle factors, such as physical activity, influence the development of diseases by separating direct effects from indirect ones mediated through cytokine pathways.

In this research, we apply MR mediation to explore the causal relationship between physical activity and CVD risk, with a particular focus on the role of circulating cytokines. Specifically, we aim to (1) determine whether genetically predicted physical activity levels protect against CAD, HF, and IHD, and (2) evaluate how circulating cytokines mediate this relationship. Our findings could contribute to a more comprehensive comprehension of the biological pathways by which physical activity affects cardiovascular health, offering insights for the development of targeted prevention strategies through lifestyle changes and anti-inflammatory interventions.

## 2. Results

### 2.1. Selection of IVs

We identified a series of single nucleotide polymorphisms (SNPs) associated with physical activity using genome-wide association study (GWAS) data. To ensure the validity of our instruments, we excluded SNPs strongly correlated with confounders or CVD outcomes (*p* < 5 × 10^−8^). Specifically, SNP rs9293503 was excluded due to its significant association with CVD, which could confound the causal pathway.

After removing potential confounders, we retained seven SNPs as IVs for predicting physical activity. These SNPs were chosen based on their strong association with physical activity and their genome-wide statistical significance in the GWAS data. All chosen SNPs exhibited F-statistics greater than 10, confirming their robustness and suitability for use in the MR analysis (Supplementary Table S2).

### 2.2. Causal Association Between Physical Activity and Cardiovascular Diseases

The main analysis using inverse-variance weighted (IVW) methods revealed a significant inverse association between genetically predicted physical activity and IHD (β = −0.053, 95% confidence interval [CI]: −0.097 to −0.009; *p* = 0.018). This indicates that higher levels of physical activity could reduce the risk of IHD. Further analysis by MR-Egger regression and the weighted median method confirmed these findings, showing consistent effect directions.

In contrast, no statistically significant associations were found between physical activity and CAD or HF, as these outcomes failed to achieve statistical significance in the analysis (Supplementary Table S3). These results imply that physical activity may specifically reduce the risk of IHD, but its impact on other forms of CVD, such as CAD and HF, remains uncertain.

To validate the robustness of our results, we conducted additional sensitivity analyses. Cochran’s Q test showed no significant heterogeneity among the selected genetic instruments (*p* > 0.05), confirming their consistency. The MR-Egger intercept test indicated no signs of horizontal pleiotropy (*p* > 0.05), further supporting the robustness of the causal estimates. Additionally, the MR-PRESSO global test did not detect any outlier SNPs, and leave-one-out analyses confirmed that no individual SNP significantly influenced the causal estimates (Supplementary Table S4 and Figures S1–S3).

### 2.3. Mediation MR Analyses of Circulating Cytokines

We conducted a two-step MR analysis to explore how circulating cytokines mediate the link between physical activity and CVDs. In the initial phase, we identified the cytokines that are influenced by physical activity, using MR on GWAS data related to both physical activity and cytokines (Supplementary Table S5).

**Figure 1:**
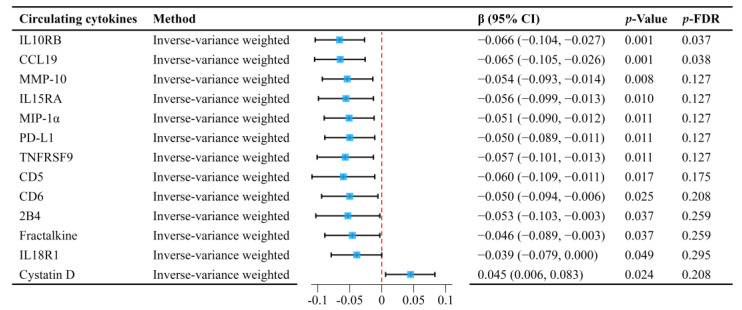
MR analyses show causal effects of physical activity on significant circulating cytokines.

Preliminary analyses, without correction for false discovery rate (FDR), showed that physical activity had a significant impact on multiple circulating cytokines. Specifically, physical activity was linked to decreased levels of multiple cytokines, including Interleukin-10 receptor subunit beta (IL10RB), C-C motif chemokine 19 (CCL19), and Matrix metalloproteinase-10 (MMP-10), among others. Conversely, Cystatin D levels were increased (Figure 1). After applying FDR correction, the relationship between physical activity and CCL19 (β = −0.065, 95% CI: −0.105 to −0.026; *p* = 0.038) and IL10RB (β = −0.066, 95% CI: −0.104 to −0.027; *p* = 0.037) remained statistically significant. Sensitivity analyses provided additional confirmation of the robustness of these findings, as detailed in Supplementary Table S4 and Figures S4–S6.

During the second phase, we examined the causal link between the cytokines affected by physical activity and CVDs. Due to the scarcity of SNPs meeting the standard *p* < 5 × 10^−8^ significance threshold, we increased the threshold to *p* < 5 × 10^−7^ to identify SNPs associated with CCL19 and IL10RB. Details regarding these SNPs are outlined in Supplementary Table S6, with F-statistics confirming no weak instrument bias.

The primary IVW analysis demonstrated a significant causal association between IL10RB levels and IHD (β = 0.054, 95% CI: 0.010 to 0.097; *p* = 0.015), suggesting that higher levels of this cytokine may increase the risk of IHD. This result was further corroborated by supplementary MR-Egger regression and weighted median analyses, which confirmed the stability of the effect direction. However, no significant relationship was observed between CCL19 levels and IHD (Supplementary Table S7). Additional sensitivity analyses validated the stability of these results (Supplementary Table S4 and Figures S7–S9).

Lastly, we assessed the mediating role of IL10RB levels in the association between physical activity and IHD. Using the causal estimates derived from the previous analyses, we calculated that approximately 6.65% of the impact of physical activity on IHD was mediated via changes in IL10RB levels.

## 3. Discussion

This research offers important perspectives on the causal relationship between physical activity and CVD risk, particularly examining circulating cytokines as potential mediators. By using MR, we established that higher genetically predicted physical activity was associated with a reduced risk of IHD. Through a two-step MR analysis, we identified IL10RB as a partial mediator in this beneficial outcome, highlighting the immunomodulatory pathways through which physical activity influences cardiovascular health. These results highlight how physical activity may help reduce CVD risk through immune modulation, particularly via IL10RB signaling, and may provide insights into future intervention strategies.

Our initial MR analysis demonstrated a significant negative association between genetically predicted physical activity and IHD, consistent with extensive evidence linking physical activity to reduced CVD risk. Regular physical activity is well known to exert cardiovascular protective effects by improving metabolic profiles, enhancing endothelial function, reducing inflammation, and promoting overall cardiovascular health [17,18]. However, our study uniquely isolates the impact of physical activity on IHD, while no significant associations were found for CAD or HF. This implies that the positive impacts of physical activity may be more pronounced for certain CVDs, particularly those driven by atherosclerotic processes like IHD. These findings highlight the need for further investigation into how physical activity impacts different cardiovascular conditions, as the underlying pathophysiology of CAD and HF may involve separate inflammatory or metabolic pathways.

A novel aspect of this study is the confirmation of IL10RB as a partial mediator between physical activity and IHD. Initially, several cytokines showed significance in our preliminary analyses. However, after applying FDR correction, only IL10RB and CCL19 remained statistically significant. Importantly, IL10RB was the only cytokine to exhibit a significant causal relationship with IHD, whereas CCL19 did not. IL10RB is a key component of the Interleukin-10 (IL-10) receptor complex, a potent anti-inflammatory cytokine critical for regulating immune responses and modulating inflammation in atherosclerosis and endothelial dysfunction [19]. Reduced expression of IL10RB may enhance anti-inflammatory effects, leading to improved immune surveillance and a reduction in the inflammatory burden associated with atherosclerosis and endothelial dysfunction. These processes are key contributors to IHD risk, suggesting that IL10RB modulation could influence cardiovascular health through immune regulation. IL-10 has been implicated in a variety of inflammatory processes, including modulation of atherosclerosis, endothelial dysfunction, and immune responses to vascular injury [20].

The association between higher physical activity and reduced IL10RB levels suggests that exercise may influence immune function through IL-10 signaling, enhancing immune surveillance, reducing inflammation, and potentially slowing atherosclerotic progression, a key factor in IHD development [21]. However, while these observations are promising, the precise mechanistic pathways linking IL10RB expression with decreased IHD risk are still not fully understood, and further basic experimental studies are needed to validate this pathway. It is crucial to investigate how IL10RB expression influences immune responses at the molecular and cellular level, particularly how it impacts inflammatory mediators that drive the pathogenesis of atherosclerosis and endothelial dysfunction.

The partial mediation effect observed through IL10RB, accounting for 6.65% of the connection between physical activity and IHD, while statistically significant, suggests that immune modulation may contribute to the cardiovascular benefits of exercise. However, given the modest size of this effect, it is important to interpret its clinical relevance cautiously. This suggests that while IL10RB is a potential therapeutic target, its role alone may not be sufficient to substantially reduce cardiovascular risk. Other factors, such as the broader network of cytokines and non-immune mechanisms, may also play significant roles in influencing cardiovascular health. These mechanisms may include not only cytokine modulation but also improvements in lipid profiles, reduced oxidative stress, and better vascular health [22]. The modest mediation effect by IL10RB suggests that while immune modulation plays a role, other non-immunological pathways, such as metabolic adaptations, improved vascular function, and hormonal changes, are likely to be involved as well [23]. This highlights the multifactorial nature of the cardiovascular benefits of exercise and underscores the need for a holistic understanding of how physical activity affects heart health.

Furthermore, our findings emphasize the complexity of cytokine signaling in CVD. The role of IL-10 and its receptor in cardiovascular health is multifaceted and context-dependent. While IL-10 is generally considered protective due to its anti-inflammatory effects, it can also exhibit pro-inflammatory actions in certain conditions [24]. This dual nature suggests that cytokine signaling is highly contextual, and changes in IL-10 levels could have different effects depending on the stage of disease progression or the specific inflammatory environment. The observed reduction in IL10RB levels may, therefore, serve to enhance anti-inflammatory responses, improving immune surveillance and reducing the inflammatory burden on the vascular system [25]. This mechanism links physical activity with the modulation of systemic inflammation, a crucial factor in the development of IHD.

Notably, the modulation of IL10RB presents promising therapeutic potential. IL-10 therapy has shown promise in clinical trials for its ability to reduce inflammation and improve cardiovascular health. Targeting IL10RB could amplify these effects, offering a potential strategy to complement physical activity in preventing and treating cardiovascular diseases. Our results suggest that lifestyle interventions, such as exercise, could naturally influence this pathway, further opening the door for combining physical activity with immunotherapy to enhance cardiovascular outcomes.

Further validation of the expression and diagnostic value of circulating cytokines in animal models and clinical samples is warranted to confirm the clinical relevance of the IL10RB-mediated effects and strengthen the translational impact of this research. Such efforts would provide stronger evidence for the role of IL10RB and other cytokines in cardiovascular disease prevention, offering valuable targets for future therapeutic strategies.

A key strength of this study is its application of MR, which uses genetic variants as IVs to establish causal relationships while minimizing confounding and reverse causation biases commonly present in observational studies. By utilizing a two-sample MR approach, we had access to large independent datasets from GWAS, which increased statistical power and generalizability. To verify the reliability of our findings, we conducted sensitivity analyses, including Cochran’s Q test, MR-Egger regression, and MR-PRESSO. These tests confirmed the consistency of the causal relationships and showed that pleiotropy and outlier SNPs had no impact on the results.

However, there are several limitations that should be acknowledged. Although we relaxed the *p*-value threshold to increase instrument availability, this approach may introduce the risk of false positives and weak instrument bias. Additionally, since the majority of the data in this study were derived from individuals of European descent, this could restrict the applicability of our findings to other populations. Future studies should replicate these findings in a broader range of populations to evaluate their relevance across different ethnic groups, as ethnic differences may influence cytokine signaling and physical activity behaviors. It is also important to examine whether the same mediating pathways, particularly those involving IL10RB, are consistent across populations with different genetic backgrounds and lifestyle factors. Furthermore, while the 6.65% mediation effect through IL10RB is statistically significant, it remains relatively small, highlighting the need for further exploration of other mediators and pathways. Specifically, additional research should focus on other cytokines and non-immune mechanisms, including metabolic pathways, lipid profiles, and vascular health, to fully understand how physical activity influences cardiovascular risk. Such research will offer a more holistic understanding of the cardiovascular benefits of exercise.

Our study provides important public health implications, as it suggests that promoting physical activity could be an effective strategy to reduce IHD risk, not only through direct effects on cardiovascular health but also by modulating immune function. These results emphasize the potential of exercise as an intervention to reduce systemic inflammation and improve cardiovascular outcomes. Furthermore, identifying IL10RB as a mediator opens new avenues for research into cytokine-based therapies that could mimic the protective effects of exercise, offering additional strategies for preventing IHD.

## 4. Materials and Methods

### 4.1. Study Design

**Figure 2:**
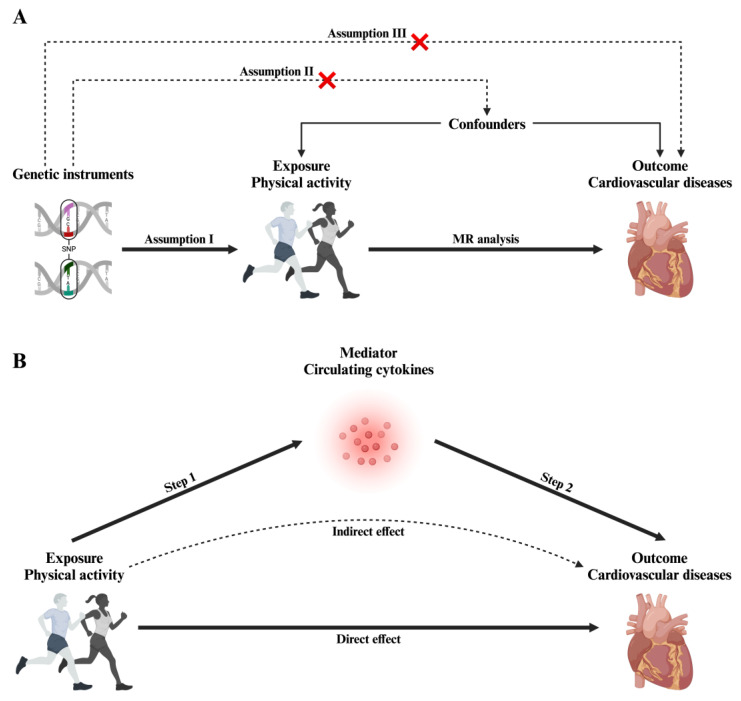
Overview of the study design. (**A**) MR analysis with three core assumptions. (**B**) Two-step MR mediation framework.

We applied a two-sample MR framework to assess the causal impact of physical activity on CVDs and to explore how circulating cytokines mediate this association (Figure 2). This approach is based on three core assumptions: (i) the genetic instruments are strongly linked with the exposure, (ii) these instruments are free from confounding factors, and (iii) they affect CVD outcomes solely through physical activity, without involving other pathways [26,27,28].

In the first phase of the analysis, we estimated the direct causal impact of physical activity on CVD outcomes using genetic variants as IVs (Figure 2A). Subsequently, we performed a two-step MR mediation analysis: first, we evaluated the causal impact of genetically predicted physical activity on circulating cytokine levels, and second, we explored how alterations in cytokine levels may influence CVD risk (Figure 2B). This sequential approach enabled us to differentiate the overall effect of physical activity on CVD into both direct and indirect pathways mediated by cytokines.

To maintain transparency and uphold methodological rigor, we followed the STROBE-MR guidelines [29], with further details available in Supplementary Table S1.

### 4.2. Data Sources

The data for physical activity were sourced from the UK Biobank cohort, which includes a total of 91,105 participants [30]. The UK Biobank is an extensive biomedical resource that includes anonymized genetic, lifestyle, and health data from approximately half a million individuals. Physical activity was measured using the Axivity AX3 wrist-worn triaxial accelerometer over a 7-day period, with raw data processed for accuracy.

Summary statistics for CVD outcomes came from large-scale genetic consortia: For CAD, we used data from the CARDIoGRAMplusC4D Consortium, which includes 122,733 cases and 424,528 controls [31]. For HF, data from the HERMES Consortium were utilized, comprising 47,309 cases and 930,014 controls [32]. For IHD, we used data from the FinnGen Consortium, including 37,854 cases and 222,551 controls [33].

Data for circulating cytokines were retrieved from a large genome-wide protein quantitative trait locus (pQTL) study that mapped 91 cytokines using the Olink Target platform. This dataset derived from 11 cohorts, encompassing 14,824 participants, with sample identifiers ranging from GCST90274758 to GCST90274848 [34].

To mitigate potential population stratification bias, all datasets used in our study involved individuals of European descent. The data were sourced from distinct cohorts, ensuring the robustness of the MR analysis. The original studies obtained the necessary ethical approval and informed consent from participants. As this study utilized only publicly available summary-level data, no additional ethical clearance was required for this analysis.

### 4.3. Selection of Genetic Instruments

We selected SNPs that showed strong associations with physical activity and cytokine level. Initially, we focused on SNPs that met a strict genome-wide significance threshold of *p* < 5 × 10^−8^ [35]. When fewer SNPs were identified, we loosened the threshold to *p* < 5 × 10^−7^ to guarantee an adequate number of instruments [36].

To avoid correlations between SNPs that could introduce bias, we applied linkage disequilibrium (LD) clumping using the PLINK algorithm, setting an r^2^ threshold of 0.001 within a 10,000 kb window [37]. Ambiguous palindromic SNPs were excluded to prevent misinterpretation. Additionally, we excluded SNPs strongly associated with CVD outcomes (*p* < 5 × 10^−8^) to prevent reverse causation [38].

The remaining SNPs were checked against the PhenoScanner database to exclude variants correlated with confounders (*p* < 5 × 10^−8^) [39]. Finally, the F-statistics for all SNPs were computed, and only those with an F-statistic exceeding 10 were retained, confirming the robustness and suitability of the selected genetic instruments [40].

### 4.4. Statistical Analysis

To obtain the primary causal estimates, we applied the IVW method, which is the most effective estimator when no pleiotropy is present [41]. To validate the results, we complemented the IVW method with MR-Egger regression and the weighted median method [42,43]. A *p*-value below 0.05 in the IVW method, coupled with consistent effect directions across methods, indicated a statistically significant causal relationship [44].

To enhance the reliability of our findings, we conducted sensitivity analyses. Cochran’s Q test was used to assess heterogeneity among the SNP-specific causal estimates, and the MR-Egger intercept test was employed to detect horizontal pleiotropy [45]. The MR-PRESSO global test was applied to correct for outlier SNPs, and leave-one-out analyses validated that no single SNP exerted an excessive influence on the causal estimates [46].

For the mediation analysis, we calculated the indirect effect of physical activity on CVD via cytokines by multiplying the effect of physical activity on cytokines (β_1_) by the effect of cytokines on CVD (β_2_) and dividing by the total effect of physical activity on CVD (β_3_) [47,48]. To account for multiple comparisons, we used the Benjamini–Hochberg FDR correction, defining statistical significance as an adjusted *p*-value below 0.05 [49]. All analyses were conducted using R (version 4.5.0) with the TwoSampleMR (version 0.6.15) and MR-PRESSO (version 1.0) packages.

## 5. Conclusions

To conclude, this study adds to the growing body of evidence supporting the beneficial effect of physical activity in reducing cardiovascular risk, highlighting the critical role of immune modulation, particularly through IL10RB. Future research is needed to confirm these results in larger and more varied populations, as well as to explore other potential mediators that could contribute to the cardiovascular benefits of physical activity. These investigations will aid in crafting more customized and effective strategies for the prevention and management of cardiovascular diseases.

## Data Availability

All data used in this study are publicly available. Summary statistics for physical activity are provided by the GWAS conducted by Klimentidis et al. [30], which can be accessed at https://sites.arizona.edu/arizonageneticepidemiology/data/ (accessed on 11 February 2025). Summary statistics for coronary artery disease are available in the GWAS by van der Harst et al. [31], accessible at https://gwas.mrcieu.ac.uk/datasets/ebi-a-GCST005195/ (accessed on 11 February 2025). Summary statistics for heart failure can be found in the GWAS by Shah et al. [32], available at https://gwas.mrcieu.ac.uk/datasets/ebi-a-GCST009541/ (accessed on 11 February 2025). Data for ischemic heart disease are sourced from the FinnGen consortium and can be downloaded at https://finngen.gitbook.io/documentation/data-download (accessed on 11 February 2025). Summary statistics for circulating cytokines are available from the GWAS by Zhao et al. [34], accessible at https://www.ebi.ac.uk/gwas/publications/37563310 (accessed on 11 February 2025). Key software packages and analysis code are available from the following repositories: https://cran.r-project.org/web/packages/MendelianRandomization/index.html (accessed on 11 February 2025), https://github.com/MRCIEU/TwoSampleMR (accessed on 11 February 2025), and https://github.com/rondolab/MR-PRESSO (accessed on 11 February 2025).

## Supplementary Materials

The following supporting information can be downloaded at: https://www.mdpi.com/article/10.3390/ijms26104615/s1.

## Abbreviations

The following abbreviations are used in this manuscript:

CCL19	C-C motif chemokine 19
CVDs	Cardiovascular diseases
CI	Confidence interval
CAD	Coronary artery disease
FDR	False discovery rate
GWAS	Genome-wide association study
HF	Heart failure
IL-10	Interleukin-10
IL10RB	Interleukin-10 receptor subunit beta
IL15RA	Interleukin-15 receptor subunit alpha
IVs	Instrumental variables
IVW	Inverse-variance weighted
LD	Linkage disequilibrium
MMP-10	Matrix metalloproteinase-10
MR	Mendelian randomization
pQTL	Protein quantitative trait locus
SNPs	Single nucleotide polymorphisms
IHD	Ischemic heart disease
