# Supplementary material for: Circulating Cytokines Mediate the Protective Effect of Physical Activity on Cardiovascular Diseases: A Mendelian Randomization Mediation Analysis
Source: Int J Mol Sci. 2025 May 12;26(10):4615. doi: 10.3390/ijms26104615 (PMC12111116; doi:10.3390/ijms26104615)
Supplement: Supplementary file 1 [file ijms-26-04615-s001.zip › Supplementary_Figures.pdf]

Supplementary Figures

Figure S1. Scatter plots of significant estimates from genetically predicted physical activity on ischemic heart disease.

Figure S2. Funnel plots of significant estimates from genetically predicted physical activity on ischemic heart disease.

Figure S3. Leave-one-out plots of significant estimates from genetically predicted physical activity on ischemic heart disease.

Figure S4. Scatter plots of significant estimates from genetically predicted physical activity on (a) IL10RB levels; (b) CCL19 levels.

Figure S5. Funnel plots of significant estimates from genetically predicted physical activity on (a) IL10RB levels; (b) CCL19 levels.

Figure S6. Leave-one-out plots of significant estimates from genetically predicted physical activity on (a) IL10RB levels; (b) CCL19 levels.

Figure S7. Scatter plots of significant estimates from genetically predicted IL10RB levels on ischemic heart disease.

Figure S8. Funnel plots of significant estimates from genetically predicted IL10RB levels on ischemic heart disease.

Figure S9. Leave-one-out plots of significant estimates from genetically predicted IL10RB levels on ischemic heart disease.

a.

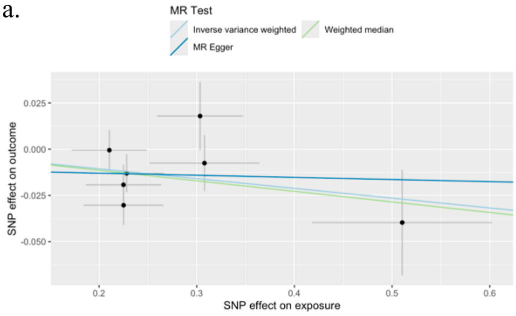

Figure S1. Scatter plots of significant estimates from genetically predicted physical activity on ischemic heart disease.

a.

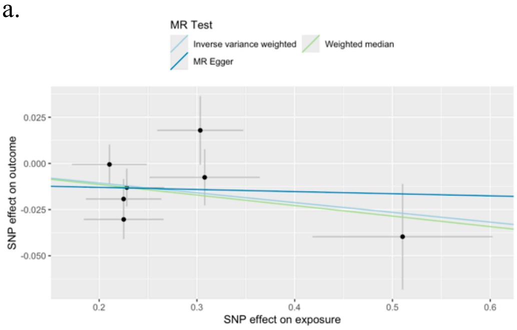

Figure S2. Funnel plots of significant estimates from genetically predicted physical activity on ischemic heart disease.

a.

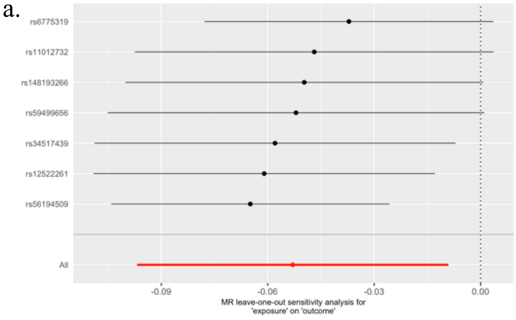

Figure S3. Leave-one-out plots of significant estimates from genetically predicted physical activity on ischemic heart disease.

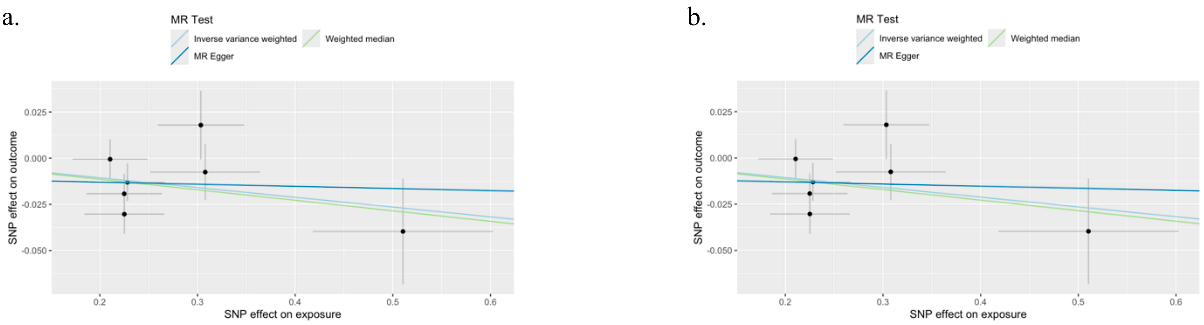

Figure S4. Scatter plots of significant estimates from genetically predicted physical activity on (a) IL10RB levels; (b) CCL19 levels.

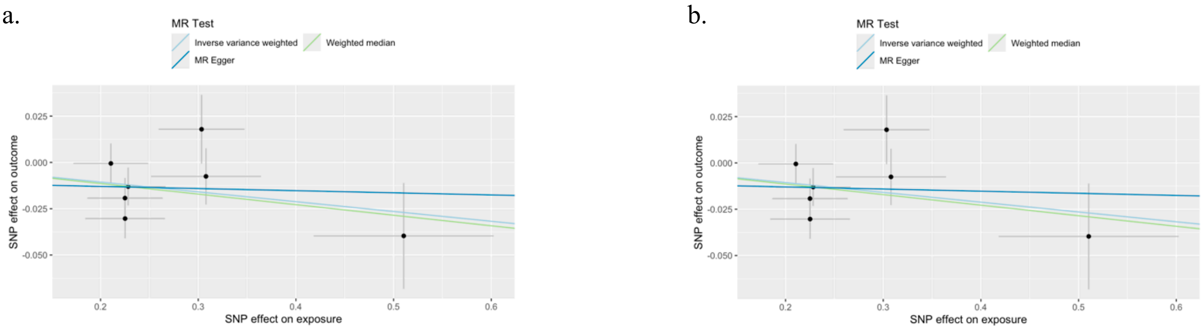

Figure S5. Funnel plots of significant estimates from genetically predicted physical activity on (a) IL10RB levels; (b) CCL19 levels.

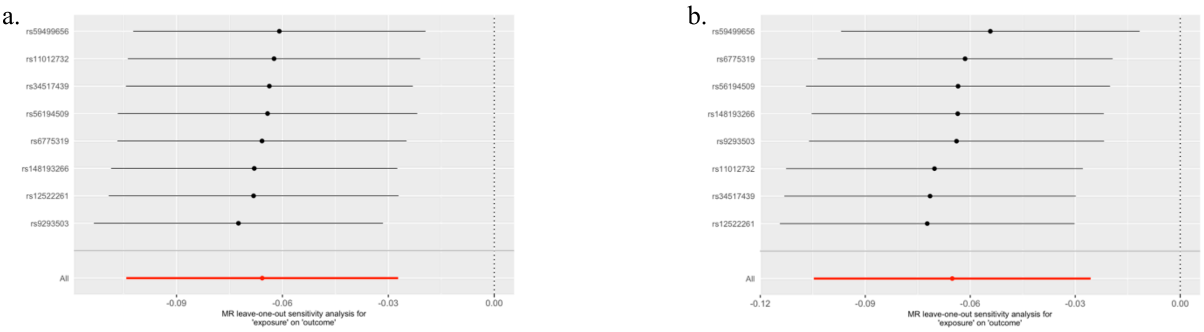

Figure S6. Leave-one-out plots of significant estimates from genetically predicted physical activity on (a) IL10RB levels; (b) CCL19 levels.

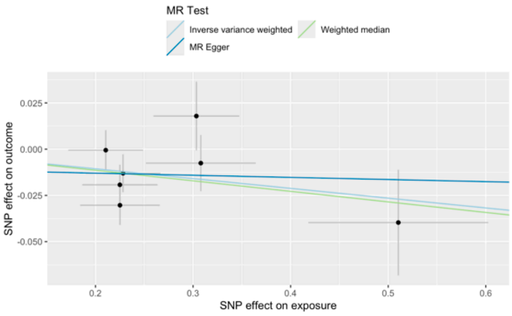

Figure S7. Scatter plots of significant estimates from genetically predicted IL10RB levels on ischemic heart disease.

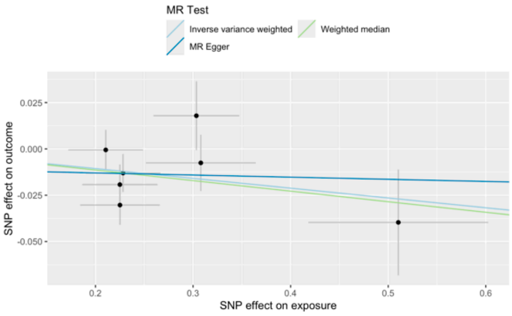

Figure S8. Funnel plots of significant estimates from genetically predicted IL10RB levels on ischemic heart disease.

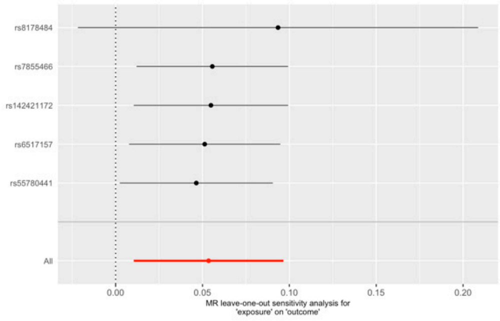

Figure S9. Leave-one-out plots of significant estimates from genetically predicted IL10RB levels on ischemic heart disease.
